# Moskowitz’s Arterial and Venous Variants (Mo Type 2) in Sigmoid Resection for Diverticular Disease: A Case Report and Literature Review

**DOI:** 10.7759/cureus.99568

**Published:** 2025-12-18

**Authors:** Antonio Costanzo, Valentina Rampulla, Antonio Varricchio, Chiara Vescovi, Lorenzo Vescovi

**Affiliations:** 1 General and Emergency Surgery, Azienda Socio Sanitaria Territoriale (ASST) Bergamo Est, Seriate, ITA; 2 General Surgery, Azienda Socio Sanitaria Territoriale (ASST) Bergamo Ovest, Ospedale di Treviglio-Caravaggio, Treviglio, ITA; 3 General and Oncological Surgery, Azienda Ospedaliera di Rilievo Nazionale (AORN) San Pio di Benevento, Benevento, ITA; 4 Human Pathology of Adult and Developmental Age "Gaetano Barresi", Università degli Studi di Messina, Messina, ITA

**Keywords:** colon and rectal surgery, diverticular disease of the colon, inferior mesenteric artery, moskowitz’s artery, venous variants

## Abstract

Diverticular disease of the sigmoid colon is common in Western countries, and acute diverticulitis may lead to complications requiring surgical intervention. Vascular anatomical variants, such as Moskowitz’s artery and inferior mesenteric vein variations, can significantly influence surgical planning and outcomes. We report the case of a 57-year-old male with acute sigmoid diverticulitis complicated by a pericolic abscess. CT imaging revealed the presence of Moskowitz’s artery and a venous variant of the inferior mesenteric vein draining directly into the portal vein. Due to clinical deterioration, a laparoscopic sigmoidectomy with protective ileostomy was performed. Intraoperatively, the arterial and venous variants were confirmed. The inferior mesenteric artery was ligated distal to the origin of the left colic artery, and the inferior mesenteric vein was ligated below the ligament of Treitz. The postoperative course was uneventful, and follow-up demonstrated good bowel function and quality of life. This case highlights the importance of recognizing vascular variants during sigmoid resection. Moskowitz’s arterial and venous anomalies may complicate dissection and increase the risk of ischemia or bleeding. Preoperative imaging and intraoperative vigilance are essential to minimize complications. Current evidence favors low ligation of the inferior mesenteric artery in diverticular disease to preserve perfusion, although the optimal strategy must be tailored to individual anatomy. Awareness of vascular variants is critical in the surgical management of diverticular disease. Integrating modern imaging techniques and individualized vascular ligation strategies enhances safety and improves outcomes in sigmoid resection.

## Introduction

Diverticular disease of the sigmoid colon is one of the most common conditions affecting the large bowel in Western countries, with prevalence increasing with age [1]. Most patients remain asymptomatic, but acute diverticulitis can lead to complications such as perforation, abscess, or fistula, requiring surgical intervention [2]. Recent randomized trials, such as the Laparoscopic Elective Sigmoid Resection Following Diverticulitis (LASER) study, emphasize selective surgery, reserving resection for complicated or recurrent cases with impaired quality of life [3].

Minimally invasive approaches, including laparoscopy and robotics, have become standard practice, improving postoperative recovery and reducing morbidity [4]. A critical aspect of sigmoid resection is the vascular anatomy of the left colon. The inferior mesenteric artery (IMA) demonstrates significant variability in its branching pattern [5,6]. Accessory branches such as Moskowitz’s artery may alter perfusion and complicate dissection if not recognized.

Similarly, the inferior mesenteric vein (IMV) shows considerable variability in its drainage, most commonly into the splenic vein but occasionally into the superior mesenteric vein or directly into the portal vein. Variants such as the “Mo type 2” highlight the importance of preoperative imaging and intraoperative awareness [7]. Another debated issue is the level of IMA ligation. High ligation at the origin facilitates mobilization but may compromise perfusion, while low ligation distal to the left colic artery preserves collateral circulation. In diverticular disease, where oncological clearance is not required, low ligation is often preferred to reduce ischemic risk and anastomotic leakage [8-10].

## Case presentation

A 57-year-old male with a history of dyslipidemia and known sigmoid diverticulosis presented to the emergency department with left iliac fossa pain. Laboratory tests revealed leukocytosis (11 ×10^9^/L), C-reactive protein (CRP) (54 mg/L), and normal renal and liver function (Table 1).

**Table 1:** Laboratory test.

Laboratory test	Value	Reference value
White blood cells	11 ×10^9^/L	4-10 ×10^9^/L
CRP (C-reactive protein)	54 mg/L	<5 mg/L
Creatinine	0.7 mg/dL	0.51-0.95 mg/dL
AST (aspartate aminotransferase)	28 UI/L	<35 UI/L
ALT (alanine aminotransferase)	32 UI/L	<35 UI/L
Sodium	140 mmol/L	136-145 mmol/L
Potassium	3.7 mmol/L	3.4-4.5 mmol/L
Total bilirubin	0.9 mg/dL	<1.20 mg/dL

**Figure 1:**
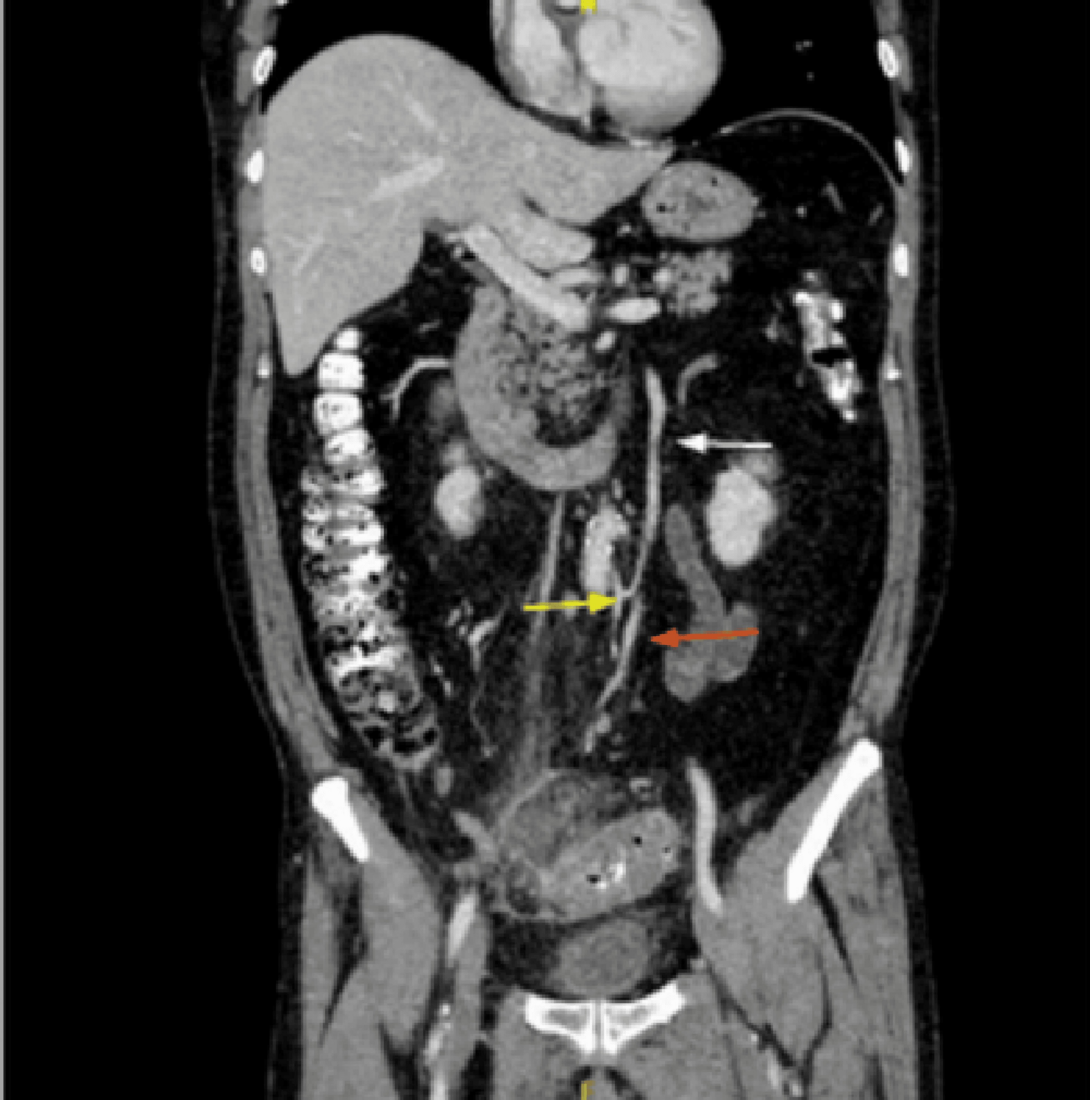
Coronal abdomen CT scan with contrast. White arrow indicates Moskowitz’s artery, orange arrow indicates inferior mesenteric vein, and yellow arrow indicates inferior mesenteric artery and its origin from aorta.

**Figure 2:**
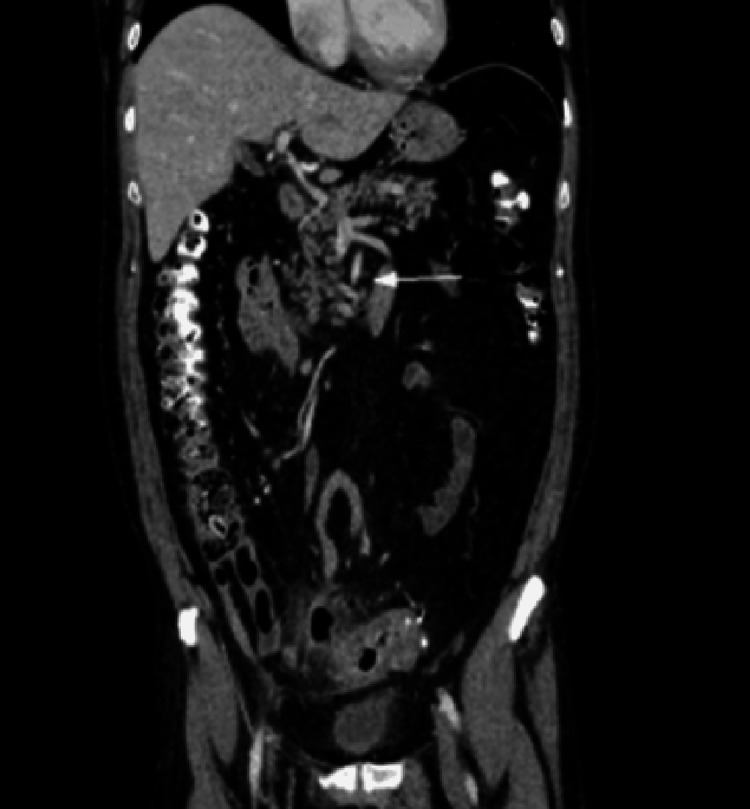
Abdomen CT scan with contrast. White arrow indicates inferior mesenteric vein without connection with the splenic vein; it flows directly into the portal vein.

Abdomen CT scan with contrast demonstrated acute sigmoid diverticulitis with a pericolic abscess measuring 5 × 3.3 cm (World Society of Emergency Surgery (WSES) stage 2A) [1]. CT imaging also revealed the presence of Moskowitz’s artery and a venous variant of the inferior mesenteric vein (IMV) draining directly into the portal vein (Figures 1, 2).

Initial empiric antibiotic therapy with piperacillin and tazobactam failed, and the patient developed peritonitis after two days of hospitalization. Laparoscopic exploration was performed, with drainage of pericolic and pelvic abscesses, sigmoidectomy, and protective loop ileostomy. Intraoperatively, Moskowitz’s artery was confirmed. The inferior mesenteric artery (IMA) was ligated distal to the origin of the left colic artery and Moskowitz and the inferior mesenteric vein (IMV) was ligated below the ligament of Treitz.

Histology confirmed diverticular disease with acute abscess formation. The postoperative course was uneventful, with surgical drainage removal on day 5 and discharge on day 7. Follow-up at 10 days, three months, nine months, and two years demonstrated normal bowel function and good quality of life.

## Discussion

Review of literature

Several studies have addressed the management of diverticular disease and the impact of vascular anatomy on surgical outcomes. The WSES guidelines provide evidence-based recommendations for acute diverticulitis, emphasizing selective surgery and the importance of CT imaging in classification [1,2]. The LASER randomized trial demonstrated that elective sigmoid resection improves long-term quality of life compared to conservative management [3].

Practice surveys, such as the Italian Society of Colorectal Surgery (SICCR) study, confirm the increasing adoption of minimally invasive approaches [4]. Anatomical studies highlight the variability of the IMA, with systematic reviews and CT angiography confirming the prevalence of accessory branches such as Moskowitz’s artery [5,6]. Similarly, atlases and radiological reviews describe IMV variants, including direct drainage into the portal vein [7,8].

The level of IMA ligation remains debated. Systematic reviews and comparative studies suggest that in diverticular disease, low ligation is preferable to preserve perfusion and reduce anastomotic complications. Editorials in 2024 reaffirm that while oncological clearance requires high ligation, diverticular disease benefits from preservation strategies [9-10].

Discussion

This case is notable for the simultaneous presence of both an arterial and a venous variants. Recognition of such anatomical variations is essential to minimize intraoperative complications and ensure adequate perfusion of the colorectal anastomosis [5-7]. In our case, ligation distal to the left colic artery was performed, consistent with institutional preference. In recent years, a trend toward ligation of the sigmoid vessels at various heights in the mesocolon has been spreading. But in this case, the risk of bleeding increases; therefore, many centers that adopt this approach perform intraoperative rectoscopy for possible endoscopic hemostasis [10]. The identification of both Moskowitz’s arterial and a venous variants draining directly into the portal vein underscores the importance of preoperative imaging and intraoperative vigilance.

## Conclusions

Sigmoid resection for diverticular disease requires not only technical expertise but also a thorough understanding of vascular anatomy. The presence of arterial and venous variants, such as Moskowitz’s artery and Mo-type IMV, can significantly impact surgical planning and outcomes. Preoperative imaging and intraoperative recognition of these variants are critical to avoid complications and ensure adequate perfusion.

In diverticular disease, low ligation of the IMA is increasingly favored to preserve vascular supply, although the optimal strategy must be individualized. This case highlights how anatomical variations directly influence surgical decision-making and reinforces the importance of integrating modern imaging techniques and anatomical awareness into routine practice.
